# Improving Ship Detection with Polarimetric SAR based on Convolution between Co-polarization Channels

**DOI:** 10.3390/s90201221

**Published:** 2009-02-25

**Authors:** Haiyan Li, Yijun He, Wenguang Wang

**Affiliations:** 1 Institute of Oceanology, Chinese Academy of Sciences, Qingdao Shandong, 266071, P.R. China; 2 College of Earth Sciences, Graduate School of Chinese Academy of Sciences, Beijing 100049, P.R. China; 3 Electronic and Information Engineer, Beihang University, Beijing 100083, P.R. China

**Keywords:** polarimetric SAR, co-polarization, ship detection, convolution

## Abstract

The convolution between co-polarization amplitude only data is studied to improve ship detection performance. The different statistical behaviors of ships and surrounding ocean are characterized a by two-dimensional convolution function (2D-CF) between different polarization channels. The convolution value of the ocean decreases relative to initial data, while that of ships increases. Therefore the contrast of ships to ocean is increased. The opposite variation trend of ocean and ships can distinguish the high intensity ocean clutter from ships' signatures. The new criterion can generally avoid mistaken detection by a constant false alarm rate detector. Our new ship detector is compared with other polarimetric approaches, and the results confirm the robustness of the proposed method.

## 1. Introduction

Ship detection by Synthetic Aperture Radar (SAR) has been widely used for monitoring fishing vessels, oil pollution, and traffic and immigration controls. There are two different fundamental modes of ship detection with single-channel (HH, HV or VV) SAR imagery: detection of the ship's wake [1-7] and detection of the ship itself [8-10]. For the former, many factors affect the imaging of wakes on SAR images, such as the state of the sea, stationary ships and radar imaging parameters; 37% of ships could not be detected using only their wake with ERS-1 and Seasat SAR data [8], and the figure is expected to be larger with RadarSAT-1 because of its lower HH-polarization signal-to-noise ratio (SNR). The latter is effective to detect ship's signatures whose intensity is larger than the threshold. However, the dimensions and shapes of ships and sea state are, *a priori*, unknown and very diverse. A reasonable threshold is the key to reduce misses and false detection. Therefore the method is conceived to discriminate an exceptionally bright localized pattern, according to an established decision rule. Otherwise, these algorithms assume the homogeneity of the statistical distribution of the image to be analyzed within a region of fixed dimensions. Owing to these limitations, researchers focus on ship detection with polarimetric SAR data.

Traditional single-polarization SAR data is not sufficient for ship detection in that it cannot fully characterize the scattering mechanisms. Many researchers have explored the use of polarimetric information for ship detection. In 1999, Ringrose and Nicola [11] first applied the coherent target decomposition (CTD) [12] method on ship detection and the scattering mechanism of each pixel was analyzed. The method was tested by a simulated image and a SIR-C/X image. In 2001, Yeremy *et al*. [13] combined the decomposition methods proposed by Cameron *et al*. [12] and Van Zyl [14], the results were satisfactory. Touzi *et al*. [15] introduced the symmetric scattering characterization method and detected two ships (35.4 m and 28.3 m) with CV-580 full polarization data. On the other hand, Touzi *et al*. [15-18] focused on polarimetric entropy, and pointed out that when wind conditions were lower than 20 knots and the incidence angle was lower 60 degrees, the application of the method on ship detection would not be limited, but entropy would degrade under rough sea conditions (>20 knots). Although these methods are effective in ship detection, they require full-polarization data. They can not be used with dual-polarization (Envisat ASAR) data, not even with only-amplitude dual-polarization data.

A somewhat similar technique of SAR interferometry for ship detection has been described by Arnaud [19]. The idea is to use information from the interferogram from two different looks processed from a single aperture SAR system. This is done by selecting two different non-overlapping bands from the synthetic aperture Doppler spectrum. Iehara *et al*. [20], Ouchi and Yaguchi [21] and Ouchi *et al*. [22] have developed Arnaud's work. They suggested taking advantage of the fact that two different looks processed from different sub-apertures of a SAR system would be separated by a small time delay. Owing to this time delay and different scattering mechanisms, there will be little correlation of the ocean backscatter in the two images but a large correlation in the backscatter from ships. Thus they proposed measuring the correlation between the looks to detect ships. Iehara *et al*. [20] computed the two-dimensional cross correlation function of the two images for ship detection, while Ouchi and Yaguchi [21] calculated the correlation with coherence. Both methods can detect ships, even when the intensity of the ship backscatter is similar to that of the surrounding ocean. However, these methods need raw data to calculate the Doppler spectrum and this degrades the resolution.

In Arnaud's work [19], the time difference is critical. His application is to the ERS satellite SAR in which case the time difference will be hundreds of milliseconds. Arnuad's presumption is that during this time delay the sea, which is continuously moving, will have strongly incoherent behaviour while ships like point scatterers will have coherent behaviour. With this method, Arnaud obtained promising results. In Ouchi's work [22], the integration time of each look is calculated as 0.28 s, and the center time difference is 0.028 s.

Inspired by this pioneerng work, we wanted to use the time different between the co-polarization. For SIR-C/X data, the pulse repetition frequency is 1,395∼1,736 Hz, the pulse length ranges from 8.5 to 40 milliseconds. SIR-C/X transmits alternating H/V with simultaneous receiving, so there is a time difference between co-polarization. Owing to this time delay and different scattering mechanisms, the ships are distinguished from the ocean cluster. The method neither needs raw data nor degrades the resolution with co-polarization data.

The two-dimensional convolution function (2D-CF) is adopted to characterize the degree of correlation between two polarization channels. The 2D-CF is extracted from the corresponding windows from each channel. If the windows contain a ship, the 2D-CF has a peak, otherwise ocean clutter. The paper is organized as follows. The next section will introduce the basic concept of convolution and the results of simulated images. In Section 3, the method is employed on a SIR-C/X SAR image. Polarimetric parameters *H*, *á*, *PD ϕ_hh−vv_*, polarmetric correlation coefficient and the results of CFAR algorithm are analyzed to confirm the results of convolution in Section 4. Another kind of polarimetric SAR data is used to validate 2D-CF in Section 5. Finally, several concluding remarks are presented in Section 6.

## 2. The theory and the analysis of simulated images

### 2.1 The convolution between co-polarization channels HH and VV

The definition of two dimensions cross correlation function (2D-CCF) is given as follows [20]:

(1) $$2D-\text{CCF}(x,y)=\iint f_{1}(X,Y)f_{2}(X+x,Y+y)dXdY$$

The definition of 2D-CF is given as follows [23]:

(2) $$2D-\text{CF}(x,y)=f_{1}⊗f_{2}=\iint f_{1}(X,Y)f_{2}(x-X,y-Y)dXdY$$

where *f*_1_(*X*, *Y*) and *f*_2_(*X*, *Y*) are the intensities of different polarimetric channels, respectively. Except for the minus sign, 2D-CF is the same as 2D-CCF. The minus sign only folds the function *f*(*X*, *Y*) to *f*_1_(−*X*, −*Y*).

Letting *X′* = −*X*, *Y′* = −*Y*, so equation (2) is equivalent to:

(3) $$2D-\text{CF}(x,y)=\iint f_{1}(X,Y)f_{2}(x+X\prime ,y+Y\prime )d(-X\prime )d(-Y\prime )$$

If *f*_1_ and *f*_2_ are both even, or both odd, then:

(4) $$2D-\text{CF}(x,y)=\iint f_{1}(X,Y)f_{2}(x+X,y+Y)d(X)d(Y)=2D-\text{CCF}(x,y)$$

It is easy to extend *f*_1_ and *f*_2_ into even or odd function. Therefore, 2*D*−*CF*(*x*, *y*) can be used to describe the cross-correlation between the co- polarization images.

For the discrete signals, equation (2) can be rewritten as:

(5) $$2D-\text{CF}(x,y)=f_{1}⊗f_{2}=\sum_{X=-\infty }^{+\infty }\sum_{Y=-\infty }^{+\infty }f_{1}(X,Y)f_{2}(x-X,y-Y)$$

Because of the time delay between the two co-polarizations, the continuously changing ocean surface will have a weak overlap behavior between the two co-polarization channels. However, the point scatters of ships have strong overlap behaviors for the different channel imaging of the same ships. Owing to the fact that 2D-CF is closely related with 2D-CCF, the different statistical behaviors of ships and ocean are also characterized by 2D-CF. Therefore, 2D-CF can be used to detect ships. If there is no overlap between polarimetric channels 1 and 2, 2D-CF is flat because it is almost the same value for the entire (*X*, *Y*). 2D-CF has a strong peak when the area contains a ship which overlaps in the images of two different channels. Thus the existence of a ship can be judged by the appearance of a peak in the 2D-CF images.

The processing flow is as follows:

1. Extract the sub-window from each channel,
2. Calculate the 2D-CF between the two co-polarization images,
3. If the 2D-CF has a peak, the sub-window is judged to contain a ship,
4. Process the entire images by moving the sub-window to the next area.

### 2.2 The results of simulative SAR images

The ocean surface is simulated on each polarimetric channel as an independent K-distribution, which has proven to be successful in many cases [24]. To preserve the polarimetric correlation of ships in HH and VV images, the ship's signatures of ship 1 for HH and VV channels in the SIR-C/X data are adapted. The difference between the real SIR-C/X ship's signature and the simulated ocean surface signature are subtracted to get the images where the return wave of ships are at a similar intensity to ocean. Then the ships' signatures of HH and VV channels are embedded into the simulated HH and VV channels ocean images, respectively.

**Figure 1.**
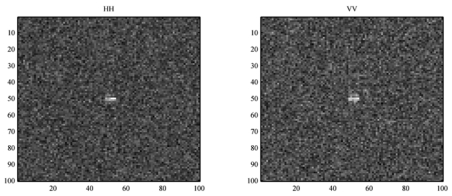
The simulated HH and VV polarization images.

In Figure 1, the contrast of ship to ocean for HH polarization is better than VV polarization. The ocean images, with different shapes, have little overlap between different polarization channels, while for ships the opposite happens. The following shows the effects of ship size, sea state and ship shape on convolution.

#### 2.2.1 The variation of ship size

**Figure 2.**
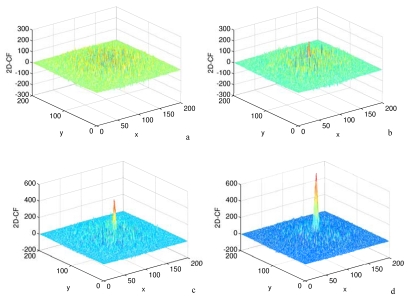
The effects of ships' size on 2D-CF. a, b, c and d are the 2D-CF values without ship signatures, the size of ship signatures are 4×4, 6×6 and 8×8 pixels, respectively. X and Y axes represent the number of pixels in range and azimuth direction. In all the following figures, the physical interpretation of X and Y axes is the same.

During the simulation, SNR is set at 1.1, which represents high sea state. The variation of ship size is described by the variation of the number of ship signatures' pixels. The effects of ships' size on convolution are shown in Figure 2. In Figures 2a and 2b, the result of convolution is not obvious until the number of ship's signatures reach 4×4 pixels. Figures 2a to 2d demonstrate that the larger the ship size the higher the convolution is, therefore large ships can be easily detected.

#### 2.2.2 The variation of sea state

**Figure 3.**
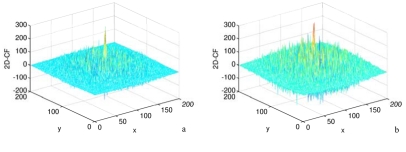
The effects of sea state on 2D-CF. a and b are 2D-CF values between co-polarization channels, SNR is 1.1 in Fig2 a. and 3.0 in Fig2 b.

During the process, the variation of SNR represents the variation of sea state. The ship size is invariable with 5×5 pixels. The higher SNR represents a lower sea state. Figures 3a and 3b show the results of different SNR values. The high value of SNR is about three times the low, which is large enough to represent different the sea state. Figure 3 shows that the convolution value is not sensitive to sea state. Therefore the method can be used under high sea state conditions (>12.5 m/s).

#### 2.2.3 The change of ships' shape

**Figure 4.**
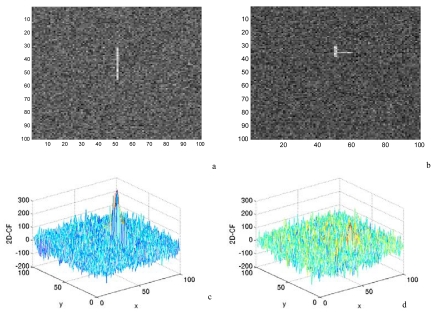
The effects of ships' shape on convolution. a and b are gray images with ‘line’ and ‘T’ shape, respectively, c and d are 2D-CF values between co-polarization channels corresponding different ship shapes.

To study the effects of ship's shape on convolution, line shape and T shape ships are embedded in the ocean clutter, respectively. Except for the shape (the array), other parameters such as the SNR and the number of pixels are the same. Figure 4 demonstrates that the peaks of 2D-CF decrease from 200 to 100 when the ship shape is changed from ‘line’ to ‘T’, which indicates the change of ships' shape obviously affects the convolution.

From above analyses one may conclude that convolution between HH and VV polarimetric channels is sensitive to ship size and shape, but not to sea state. The results benefit weak ship signature detection under higher sea state conditions. Based on the simulation, a practical ship-detection algorithm is developed.

## 3. Analysis of SIR-C/X SAR image

### 3.1 Test data description

**Figure 5.**
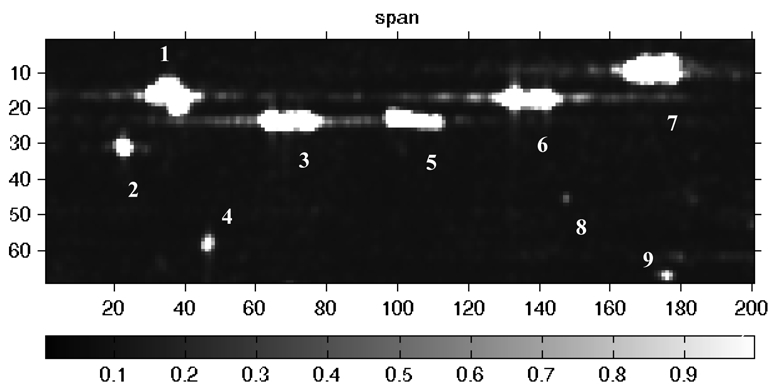
The span image of SIR-C SAR on 4^th^ Oct.1994.

To validate the performance, a full polarimetric SIR-C/X SAR image was analyzed using this method. The image was acquired on Oct 4, 1994 off Hongkong Victoria port center at E114.05° N22.106° and 33° incidence angles. The image mode is multi-look complex and the number of looks is 5.2. The sensor operated in the C band (*λ*= 5.8 cm) and the resolution was 12.5 m. The span image of the data is shown in Figure 5, in which there are many targets distinguishable from sea. They are marked with different numbers 1-9. Unfortunately, the ships were not ground-trued during SAR data acquisition, so this dataset will mainly be analyzed in terms of ship detectability.

### 3.2 The choice of window

There are many possible variations of size and shape of ships and windows and how they are moved across the image. The final choice will be determined by the detection problem at hand and will also need to take account of computational burdens. The standard practice in ship detection is to use square windows and move them as a unit. Square windows are the common choice because they allow computational efficiency and there is lack of *a priori* knowledge of preferred shapes and orientation for ships and ocean clutter. Wackerman *et al*. [10] expanded upon these topics. The sizes of the windows should be related to the sizes of ships to be detected and the resolution of the radar. The window should be a similar size to the smallest ship to be detected. Perhaps an auto-adapted, edge-aligned window is the best. However, if there is a weak target, it is difficult to ascertain the edge direction. Considering these factors and with trial and error, the size of window was 5×5 pixels and moving as a unit.

### 3.3 Application the method to the SAR image

The above image was analyzed with the method and the results are shown in Table 1. In this table, the parameters Max_b_c and Max_a_c represent the maximum power before and after convolution, respectively. In a similar way, Mean_b_c and Mean_a_c denote the mean power at the two phases. The maximal power only shows the information of one local pixel. However, it is directly related to the contrast and can provide a measurement of ship-sea ratio. The mean power will change with the numbers of pixels, but represents the general information of the object. Both parameters describe the power from different point of view. In Table 1, after convolution the max and mean power of targets 1-8 are improved, regardless of whether they are large or small,. However, for those of ocean and target 9 the very reverse ocurrs. The max power of ocean decreases from 7.4×10^-2^ in the initial data to 2.3×10^-2^ after convolution, and the mean power of the ocean decreases from 1.3×10^-2^ to 3.6×10^-3^. Similarly, the max and mean power of target 9 also reduce after convolution.

The advantages of the convolution method can be shown clearly and visually in Figure 7. The contrast of targets 1-8 has been improved owing to the act the method depressed the power of the ocean clutter and elevated that of targets. Therefore there is the potential for detecting weak ship signals with low SNR. However target 9 is an exception, its contrast has been decreased after convolution. The convolution method could improve the contrast of targets 1-8, which, we can confirm, are ships. While target 9 has the same variation trend as ocean, which is reverse to that of targets 1-8, so at first we conclude that target 9 may be ocean clutter.

## 4. Comparison with other algorithms

### 4.1 Comparison with polarimetric parameters

Without ground-truth data, we adopt the polarimetric entropy *H* and scattering angle *α*, polairmetric degree *PD* and co-polarimetric phase difference *ϕ_hh−vv_* to verify the above opinion. The following reviews these parameters simply.

Cloude and Poitter obtained the polarimetric entropy and mean alpha angle α based on the decomposition of coherence matrix [25-26]. Both parameters are widely used in classification [27-28], because they are directly related to scattering mechanisms. *H* is the measurement of randomness of the scattering mechanisms. Ocean is dominated by surface scattering with low entropy, while ships have complex scattering with correspondingly high entropy. The angle *α* stands for an indicator of the type of scattering mechanism, and it corresponds to the variation from surface scattering (*α*=0) to dipole scattering (*α*=45°) to double bounce scattering from conductive surface (*α*=90°). In fact, *PD* is the ratio between the intensity of the polarized part and the total scattered intensity [29]. The ocean is dominated by surface scattering with high polarimetric degree, while ships have complex scattering with low polarimetric degree. Just as the name describes, co-polarization phase difference *ϕ_hh−vv_*, ranging from 0° to 180°, gives the phase difference between HH and VV polarization. It has been proven to be useful in ship detection [16,30]. A single bounce scatterer generally results in a phase difference close to 0°, whereas an ideal double bounce scatterer has a phase difference of 180°. The dominant scattering mechanism of ocean is single bounce while that of ships is double bounce. All these parameters can distinguish ships in different aspects.

Figure 8 demonstrates the results of polarimetric parameters. The targets 1-8 have high *H*, *á*, *ϕ_hh−vv_* and low *PD*, which are the typical characteristics of ships. On the contrary, target 9 has low *H*, *á*, *ϕ_hh−vv_* and high *PD*, which are the same as those of ocean. Based on the features, target 9 is judged as ocean clutter. It is noticed the power of target 9 is more than ten-fold that of other ocean. The CFAR method will generally classify target 9 as a ship, in that a threshold as large as ten times that of the ocean is impossible. As expected, the convolution method has improved the contrast of targets 1-8 and decreased that of target 9. The results are consistent with those of *H*, *á*, *PD* and *ϕ_hh−vv_*, which indicates that the convolution with co-polarization amplitude data can a the same results as quad-polarization data.

### 4.2. Comparison with polarimetric correlation coefficient

The polarimetric correlation coefficient between HH and VV channels is defined by Lee *et al*. [31]:

(6) $$\rho_{\text{hhvv}}=\frac{<S_{\text{hh}}S_{\text{vv}}^{*}>}{\sqrt{<{\left|S_{\text{hh}}\right|}^{2}><{\left|S_{\text{vv}}\right|}^{2}>}}$$

where the asterisk denotes the complex conjugate. The angular brackets represent the ensemble average. Subscripts HH and VV represent the different polarimetric states. The polarimetric correlation coefficient has been used in feature extraction of polarimetric SAR data [31-32]. Liu *et al*. [30] have studied the correlation coefficients of ships and ocean for four polarimetric channels. It can distinguish ships from ocean. For ideal single-bounce or double-bounce scattering, the absolute of the correlation coefficient between HH and VV channels both are 1. For ship detection, the phase must be considered. Generally, we adopt the function:

(7) $$\rho_{\text{hhvv}}=\{\begin{array}{cc} \left|\rho_{\text{hhvv}}\right|, & \phi_{\text{hhvv}}\leq 90^{{}^{\circ}}\\ -\left|\rho_{\text{hhvv}}\right|, & \phi_{\text{hhvv}}\geq 90^{{}^{\circ}} \end{array}$$

In fact, combining the information of amplitude and phase restricts that the data type is complex. However, the convolution of co-polarization channels developed in the paper does not need the information of phase.

In Figure 9, the correlation coefficient of some ocean areas, except ships, is lower than -0.6, which will cause false detection. Comparing the correlation coefficient with 2D-CF (Figure 7), the convolution method will improve the contrast of ships to ocean with less false detection.

### 4.3. Comparison with CFAR

Detection of ships in radar clutter by calculating thresholds on clutter probability distribution functions has been extensively studied. Most of the work focuses on CFAR [8], which includes bi-parameters [10] and cell-average (CA) algorithms [33]. To compare the results of 2D-CF with CFAR, the results of bi-parameters CFAR [10] is presented.

**Figure 6.**
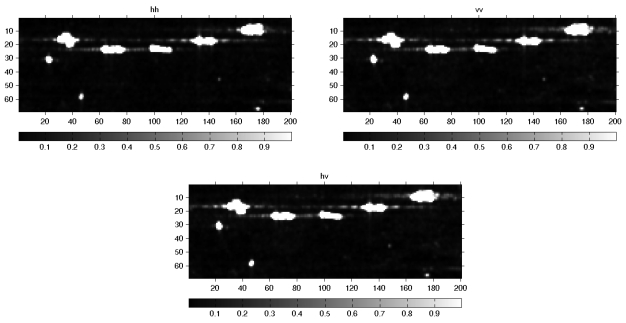
The HH,VV and HV polarization gray images of SIR-C/X data.

**Figure 7.**
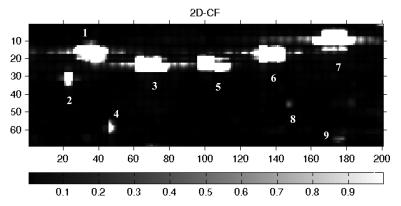
The span image after convolution. 1-9 mark the targets with higher power than ocean clutter.

**Figure 8.**
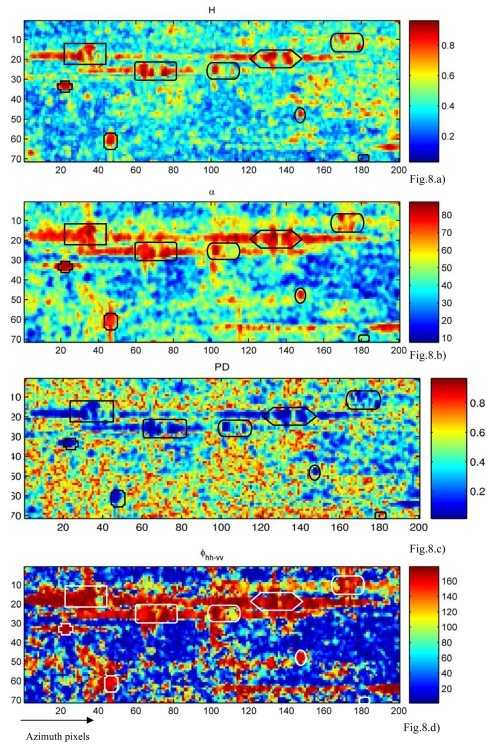
Validating the results of convolution with polarimetric parameters. a, b, c and d show the images of polarimetric entropy *H*, scattering angle *α*, polarimetric degree *PD* and co- polarimetric phase difference *ϕ_hh−vv_*.

**Figure 9.**
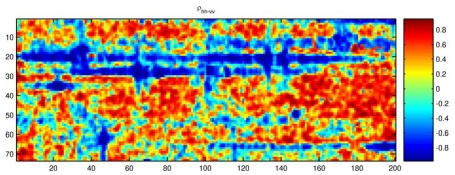
The correlation coefficient of co-polarization.

**Figure10.**
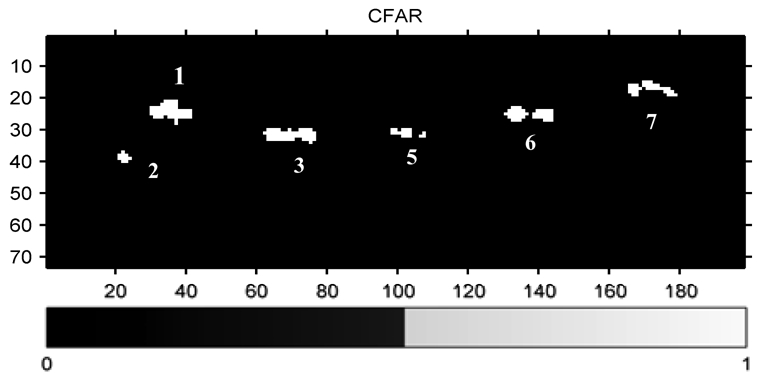
The result of CFAR.

In Figure 10, the signatures of ships 1, 2, 3, 5, 6, 7 are detected; those of weak ships 4, 8, 9 are mistaken as sea clutter. Comparing the results with 2D-CF (Figure 6), the advantages of 2D-CF are obvious.

## 5. Test of 2D-CF with AIRSAR data

**Figure 11.**
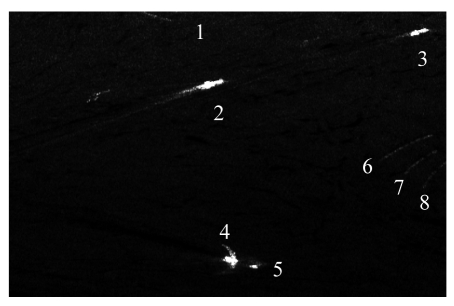
The span image of AIRSAR on 4th Oct.2000.

To test the method, a full polarimetric AIRSAR image was also analyzed. The image was acquired on Oct 4, 2000 near Japan. The center latitude and longitude is (34.37, 132.56). The sensor operated in L band. In the span image (Figure 11) there are some targets denoted with different numbers. Numbers 1, 6∼8 represent the sea bank near the island, and 2∼5 represent ships.

**Figure 12.**
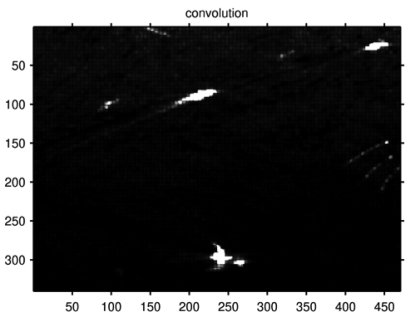
The span image after convolution of AIRSAR data.

Figure 12 shows the results of convolution with 5×5 windows, the contrast of targets 1-8 are improved, which confirms the 2-D convolution method.

## 6. Conclusions

This paper has studied ship detection with convolution between two co-polarization SAR data by applying the different statistical behavior of ships and ocean. The 2D-CF depicting the overlap and correlation of different channels is used to detect ships. Ocean is a stochastic process, so the overlap of ocean between co-polarization data is weak. On the contrary, the same ships are imaged on the co-polarization SAR images. Therefore the 2D-CF of ships will have a peak, while that of ocean is flat. The simulative processes demonstrate the size and shape of ships affect 2D-CF dramatically, while that of sea state is small. The real SIR-C/X SAR data analyses show the convolution method decreases the intensity of ocean and increases that of ship, and then enlarges the difference between ships and ocean, therefore the convolution facilitates ship detection. The opposite variation trend of ocean and ships can be considered as a new criterion. Objects which have the same polarimetric features as ocean except for high intensity can be distinguished as ocean clutter by the new criterion. While CFAR cannot make out the differences they are generally mistaken as ships. Furthermore, the results are in accord with those of polarimetric parameters, which indicate the method with co-polarization amplitude only data can reach the same effects as full-polarization data. The comparison between correlation coefficient and convolution shows that the latter improves ship detection, with less false detections. Additionally, the convolution is a bit affected with sea state and resolution, so the method facilitates ship detection in large areas under high sea state conditions. However, the convolution method will enlarge the size of ships, so it can not be used to estimate the size of ship by the number of pixels. Otherwise, the amplitude of overlap does not correspond well to the intensity of initial data, the reasons for which need further study. Improving ships detection with less data is the aim of many researchers. These encouraging results for ship detection should motivate the further study of suitable algorithms. This work will be extended to include an analysis of other datasets with different ships, different incidence angles, and different environmental conditions, to characterize performance under more general conditions.

## Figures and Tables

**Table 1.** The max power and mean power before and after convolution. The parameters Max_b_c,and Max_a_c represent the maximum of power before and after convolution, respectively. In a similar way, Mean_b_c and Mean_a_c can be referred as the mean power at two phases.

	ocean	1	2	3	4	5	6	7	8	9
Max_b_c	7.4×10^-2^	5.7×10^1^	2.7×10^1^	8.8×10^1^	2.1	5.1×10^1^	2.5×10^2^	1.4×10^2^	3.7×10^-1^	1.5
Max_a_c	2.3×10^-2^	2.0×10^4^	2.5×10^3^	1.8×10^4^	1.4×10^1^	1.2×10^3^	6.4×10^4^	4.5×10^4^	4.2×10^-1^	4.8×10^-1^
Mean_b_c	1.3×10^-2^	8.1	4.8	1.3×10^1^	7.0×10^-1^	4.9	1.4×10^1^	1. 5×10^1^	1.1×10^-1^	3.2×10^-1^
Mean_a_c	3.6×10^-3^	1.7×10^3^	4.9×10^2^	1.8×10^3^	1.8	1.2×10^2^	1.8×10^3^	5.0×10^3^	1.9×10^-1^	1.6×10^-1^
